# Abcès thyroïdien fistulisé révélant un carcinome œsophagien chez un adulte jeunee

**DOI:** 10.11604/pamj.2020.37.67.22403

**Published:** 2020-09-17

**Authors:** Carlyse Viani Diffo Siakwa, Othmane Benhoummad, Abdelaziz Raji, Mariem Ouali Idrissi, Najat Cherif Idrissi El Ganouni

**Affiliations:** 1 Service de Radiologie, CHU Mohammed VI, Université Cadi Ayyad, Marrakech, Maroc,; 2 Service d’ORL et de Chirurgie Cervico Faciale, CHU Mohammed VI, Université Cadi Ayyad, Marrakech, Maroc

**Keywords:** Abcès thyroïdien, clinique, imagerie, complications, Thyroid abscess, clinical, imaging, complications

## Abstract

L’abcès de la thyroïde est une pathologie extrêmement rare. Son diagnostic est souvent fait tardivement, ce qui prédispose à de graves complications. Nous rapportons un cas d’abcès thyroïdien chez un adulte jeune qui s’est présenté aux urgences pour une tuméfaction basi-cervicale antérieure associée à une dysphonie, une dysphagie et une dyspnée. Une échographie cervicale mettait en évidence une collection du lobe thyroïdien gauche. Un scanner cervico-thoracique objectivait un abcès thyroïdien gauche, fistulisé dans les muscles sous hyoïdiens et le sinus piriforme gauche, avec épaississement de l’hypopharynx et de la bouche œsophagienne. La ponction ramenait un liquide purulent. Une panendoscopie révélait la présence d’une prolifération tumorale de la bouche œsophagienne. Une biopsie avec étude anatomopathologique a conclu à la présence d’un carcinome épidermoïde. Cette observation révèle que l’abcès thyroïdien peut être le mode de découverte d’un cancer de l’œsophage. D’où l’intérêt de rechercher une cause sous-jacente en cas d’abcès thyroïdien.

## Abstract (en)

Thyroid abscess is an extremely rare condition. Its diagnosis is often late, which predisposes to serious complications. We report a case of thyroid abscess in a young adult who presented to the emergency room for a basi-cervical swelling associated with dysphonia, dysphagia and dyspnea. Neck ultrasound showed a collection of left thyroid lobe. A cervicothoracic computed tomography objectified an abcess of the left lobe of the thyroid fistulising in the hyoid muscles beneath and left piriform sinus with thickening of the hypopharynx and upper esophageal sphincter. The puncture brought a purulent fluid. Panendoscopy revealed the presence of a tumor proliferation of the upper esophageal sphincter. A biopsy with histological examination concluded to a squamous cell carcinoma. This observation indicates that the thyroid abscess may be the mode of discovery of esophageal cancer. Hence the interest to seek an underlying cause in cases of thyroid abscess.

## Introduction

L’abcès de la thyroïde est une pathologie extrêmement rare. Les caractéristiques anatomophysiologiques de la thyroïde lui confèrent une capacité unique de résistance aux infections [1]. Des facteurs prédisposants tels des malformations congénitales chez l’enfant ou une pathologie thyroïdienne sous-jacente chez l’adulte ont été identifiés [2]. Nous rapportons le cas d’un abcès thyroïdien fistulisé révélateur d’un carcinome œsophagien chez un jeune adulte.

## Patient et observation

**Figure 1:**
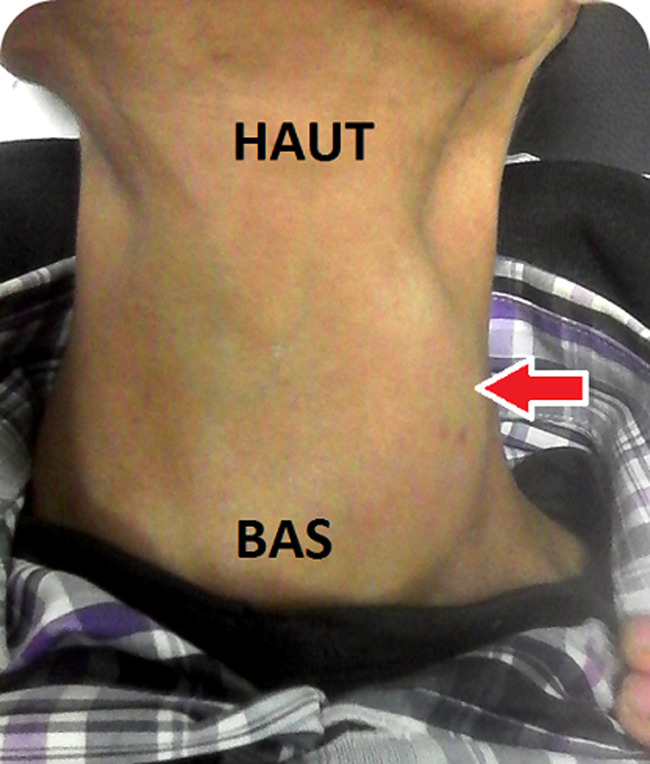
tuméfaction basi cervicale paramédiane gauche

**Figure 2:**
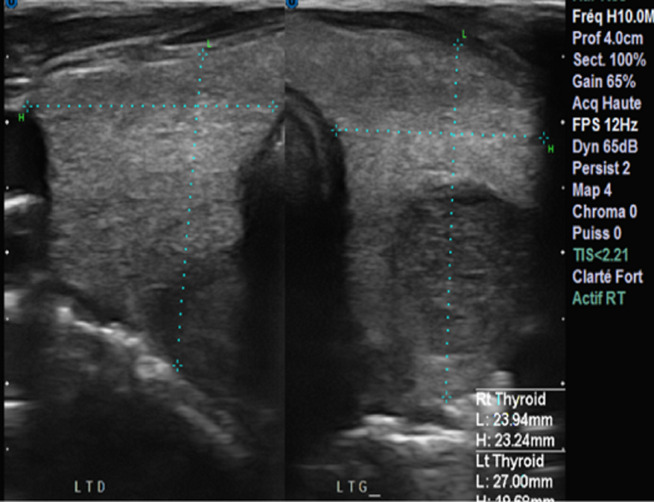
échographie thyroïdienne en coupe transversale; glande thyroïde augmentée de volume, siège d’une collection médiolobaire gauche à contenu hypoéchogène hétérogène siège de bulles d’air et de quelques calcifications, fusant vers le plan musculaire postérieur

**Figure 3:**
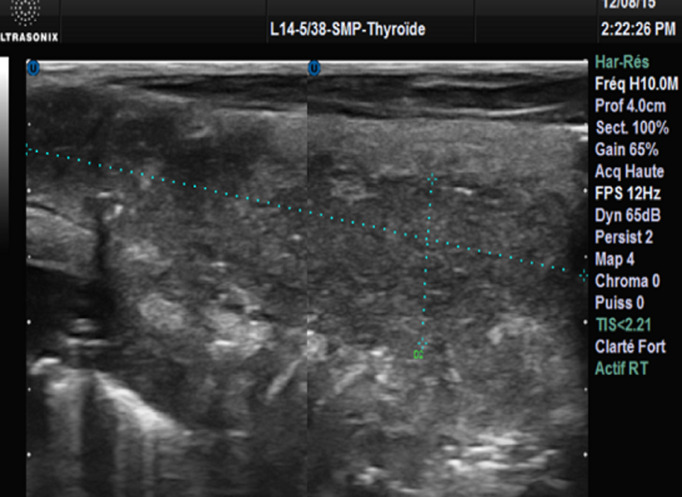
échographie thyroïdienne en coupe longitudinale; glande thyroïde augmentée de volume, siège d’une collection médiolobaire gauche à contenu hypoéchogène hétérogène siège de bulles d’air et de quelques calcifications, fusant vers le plan musculaire postérieur

**Figure 4:**
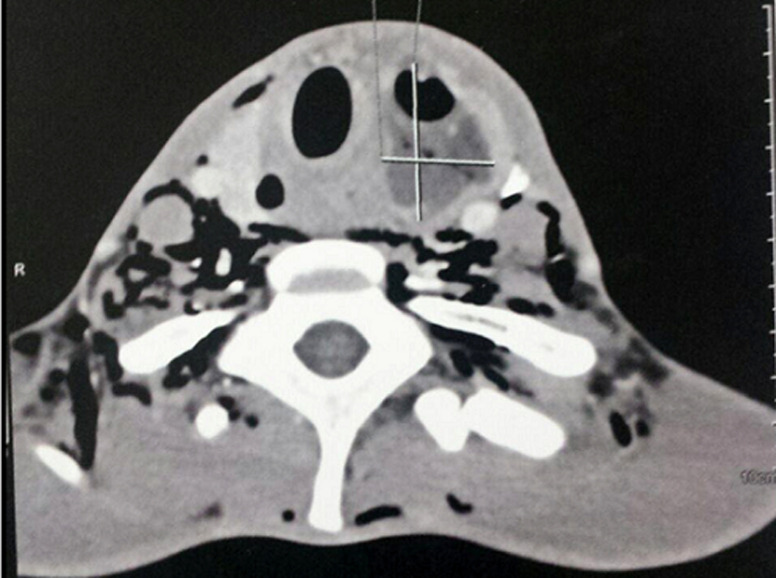
scanner cervico thoracique avec injection de produit de contraste en coupe axiale; collection liquidienne du lobe gauche de la thyroïde renfermant des bulles d’air, mesurant 50x25 mm, rehaussée en périphérie après injection de contraste, fistulisée dans les muscles sous hyoïdiens et le sinus piriforme gauche avec important emphysème cervical

**Figure 5:**
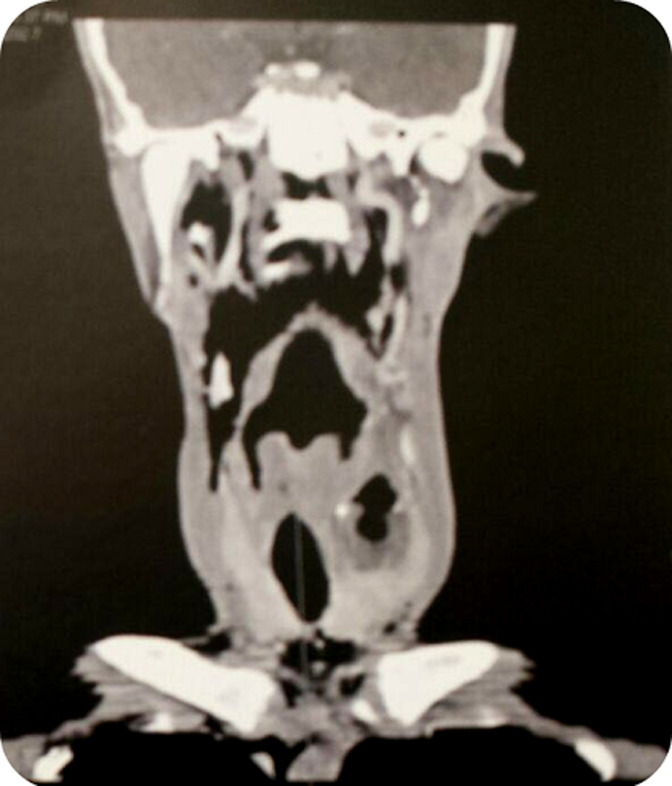
scanner cervico thoracique avec injection de produit de contraste en coupe coronale; collection liquidienne du lobe gauche de la thyroïde renfermant des bulles d’air, mesurant 50x25 mm, rehaussée en périphérie après injection de contraste, fistulisée dans les muscles sous hyoïdiens et le sinus piriforme gauche avec important emphysème cervical

**Figure 6:**
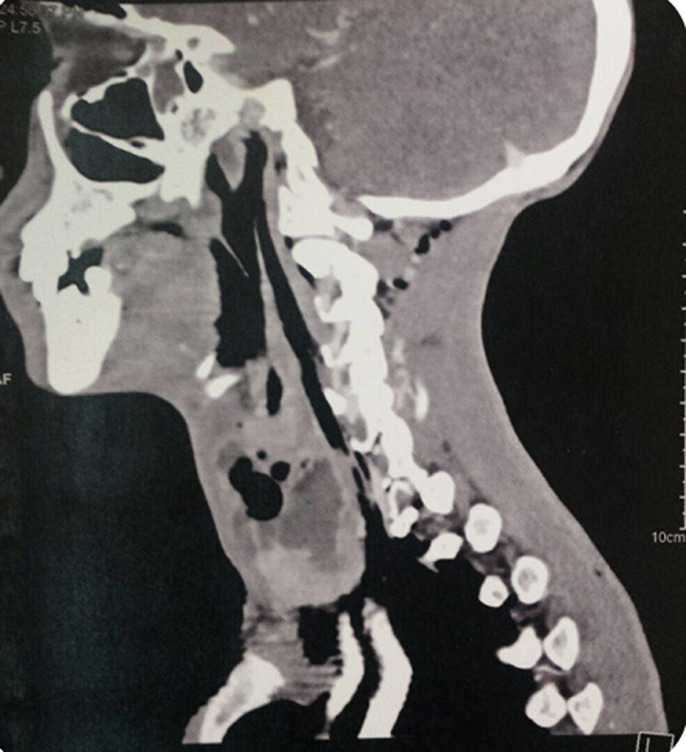
scanner cervico thoracique avec injection de produit de contraste en coupe sagittale; collection liquidienne du lobe gauche de la thyroïde renfermant des bulles d’air, mesurant 50x25 mm, rehaussée en périphérie après injection de contraste, fistulisée dans les muscles sous hyoïdiens et le sinus piriforme gauche avec important emphysème cervical

**Figure 7:**
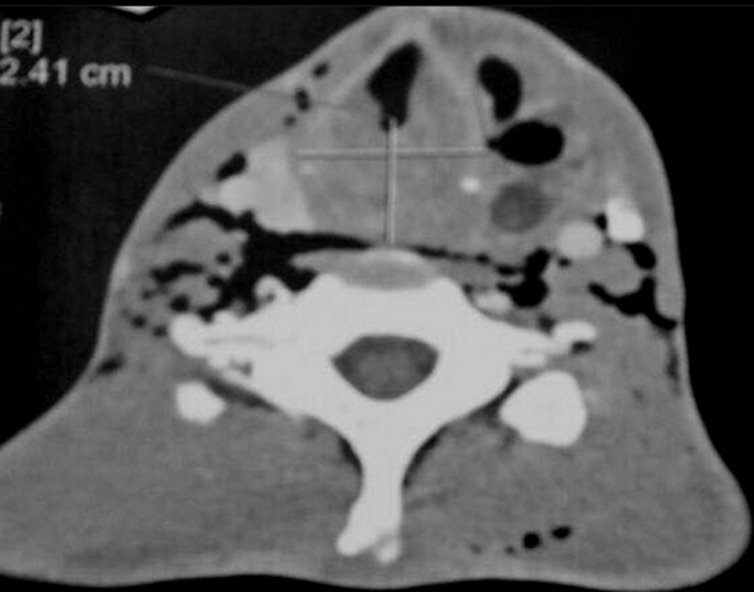
scanner cervico thoracique avec injection de produit de contraste en coupe axiale; épaississement de l’hypopharynx étendu à la bouche œsophagienne avec réduction de la filière glotto sous glottique

Il s’agissait d’un patient de 30 ans, sans antécédents pathologiques particuliers, notamment non tabagique, non alcoolique. Ce patient s’est présenté aux urgences pour une tuméfaction basi-cervicale antérieure évoluant depuis 3 mois, indolore, associée à une dysphonie, une dysphagie, une dyspnée d’aggravation progressive et un amaigrissement non chiffré, le tout évoluant dans un contexte d’altération de l’état général. A l’examen physique il était apyrétique à 37°C (avec une notion de prise antérieure d’antipyrétiques) et présentait une masse basi cervicale paramédiane gauche mobile, ascensionnant à la déglutition, de consistance molle, sans signes inflammatoires en regard (Figure 1), associée à un léger tirage sus sternal. L’hémogramme objectivait une hyperleucocytose à 11800/uL avec 77% de polynucléaires neutrophiles (PNN). La protéine C réactive (CRP) initiale était élevée à 354,1 mg/l. La TSH ultrasensible était basse, à 0,02 UI/mL. La sérologie HIV était négative. L’échographie cervicale mettait en évidence une collection thyroïdienne médio lobaire gauche, à contenu épais hypoéchogène hétérogène siège de bulles d’air et de calcifications, fusant vers le plan musculaire postérieur associée à des ganglions de voisinage d’allure inflammatoire (Figure 2, Figure 3). Le scanner cervico-thoracique objectivait une collection liquidienne du lobe gauche de la thyroïde, renfermant des bulles d’air et des calcifications, fistulisée dans les muscles sous hyoïdiens et le sinus piriforme gauche. Il s’y associait un épaississement circonférentiel de la paroi antérieure de l’hypopharynx mesurant sur le plan axial 33x24 mm, étendu à la bouche œsophagienne et aux replis ary-épiglottiques, à la commissure postérieure et la région rétro crico-thyroïdienne avec réduction de la filière glotto-sous-glottique. Le larynx était d’aspect normal, la trachée respectée. On notait quelques adénopathies jugulo-carotidiennes. Un important emphysème cervico médiastinal était également retrouvé (Figure 4, Figure 5, Figure 6, Figure 7). Le diagnostic d’abcès de la thyroïde sur carcinome pharyngo œsophagien était évoqué. La ponction ramenait du liquide purulent, confirmant le diagnostic d’abcès thyroïdien. La culture faite était positive à *Staphylococcus aureus*. Une antibiothérapie était démarrée à base d’Amoxicilline acide clavulanique, Flagyl et Gentamycine avec bonne évolution, régression de la collection au contrôle échographique et dégression de la CRP. Une nasofibroscopie objectivait des secrétions purulentes, une stase salivaire au niveau des deux sinus piriformes et une parésie des 2 cordes vocales en ouverture. Une panendoscopie sous anesthésie générale révélait la présence d’une prolifération tumorale de la bouche œsophagienne. Une biopsie faite avec étude anatomopathologique confirmait le diagnostic de carcinome épidermoïde de la bouche œsophagienne. Le patient était adressé en oncologie pour prise en charge de sa néoplasie.

## Discussion

L’abcès de la thyroïde est une entité rarement rencontrée en pratique médicale. Il ne représente que 0,1% des pathologies chirurgicales de la thyroïde [1]. En effet, la thyroïde est totalement encapsulée et isolée des organes de voisinage et est riche en iode, substance bactéricide défavorable à la prolifération bactérienne [2,3]. L’abcès thyroïdien peut survenir à tout âge [3]. Il prédomine chez les sujets jeunes, surtout ceux porteurs de pathologies thyroïdiennes pré existantes (goitre, nodule, carcinome) ou de malformations congénitales telles les fistules du tractus thyréoglosse et les fistules de la 4^e^ poche endobrachiale [1,4,5]. Le terrain immunodéprimé représente le deuxième facteur prédisposant à l’affection [4]. Notre patient n’avait pas de contexte d’immunodépression. L’abcès thyroïdien résulte le plus souvent de la dissémination hématogène à partir d’un foyer infectieux à distance. L’infection peut se faire par contiguïté, parfois suite à un traumatisme direct de la glande par un corps étranger [2,3,6]. Le *Streptococcus*, le *Staphylococcus aureus* sont les germes les plus fréquemment en cause (70%) [1,5]. D’autres espèces ont été identifiées en cas d’immunodépression [1]. L’association de l’abcès thyroïdien à un cancer de l’œsophage semble jusqu’à lors n’avoir jamais été rapportée dans la littérature. Aucun facteur favorisant sa survenue suite à un cancer de l’œsophage n’a de même été décrit.

Le cancer de l’œsophage cervical est une pathologie rare, représentant moins de 15% des cas des cancers œsophagiens. Il survient surtout chez le sujet âgé, avec un âge moyen de découverte de 60 ans [7]. Il a rarement été décrit chez le sujet jeune, comme c’était le cas pour notre patient. Le facteur de risque principal est l’intoxication alcoolotabagique, qui multiplie par 50 le risque de développer un cancer de l’œsophage [7]. Notre patient n’avait pas d’habitudes toxiques. Cliniquement, l’abcès thyroïdien se présente sous forme d’une tuméfaction cervicale douloureuse associée à une dyspnée, une dysphonie, une dysphagie et une fièvre. Le bilan biologique est souvent perturbé: augmentation de la CRP, hyperleucocytose. Le bilan hormonal est souvent normal, parfois on observe une hyper ou une hypothyroïdie [1,4]. Il est universellement reconnu que la simple ponction à l’aiguille ramenant du pus franc confirme le diagnostic d’abcès. L’étude cytobactériologique permet d’isoler l’agent microbien causal [1]. La dysphagie est le signe le plus souvent retrouvé dans la forme la plus typique du cancer de l’œsophage. Celle-ci survient classiquement d’abord aux solides puis aux liquides. À l’extrême, le patient devient aphagique. La dysphagie est liée à une obstruction d’au moins la moitié de la lumière œsophagienne, elle est donc souvent le témoin d’une lésion déjà évoluée. D’une manière générale, toute dysphagie persistante doit faire évoquer un cancer de l’œsophage, et faire pratiquer un bilan clinique et endoscopique complet, et ce d’autant plus qu’il s’agit d’un patient alcoolotabagique ou présentant d’autres facteurs de risque [7]. Les autres signes cliniques sont en lien avec une extension locorégionale de la lésion tels que la dysphonie par envahissement ou compression du nerf laryngé inférieur et la dyspnée par atteinte de la paroi trachéale postérieure ou en cas de rare paralysie récurrentielle bilatérale [7].

Dans l’abcès thyroïdien, les radiographies standards peuvent montrer une déviation de la trachée cervicale [1]. L’échographie thyroïdienne est l’examen de première intention pour le diagnostic, elle montre une image d’échostructure hypoéchogène hétérogène, unique ou multiple, uni ou bilatérale. L’échostructure de l’abcès varie en fonction de la quantité de débris internes et d’hémorragie. Il existe une vascularisation périphérique [2]. L’échographie permet aussi de détecter une anomalie thyroïdienne sous-jacente, d’éliminer une autre cause et de guider une éventuelle ponction [4]. Avec le scanner, elle est d’une aide incontournable dans l’étude de la structure de l’abcès, le nombre de logettes, sa taille et ses rapports avec les structures anatomiques adjacentes [1]. Au scanner, l’abcès se présente comme une collection liquidienne hypodense, bien limitée par une coque fibro-inflammatoire rehaussée après injection de produit de contraste. Le scanner a un intérêt limité dans l’établissement du diagnostic, il sert surtout à la recherche d’une cause locale et au bilan d’extension locorégional [4]. Après résolution de l’épisode aigu, il est utile pour rechercher des malformations congénitales telles une fistule du sinus piriforme après ingestion d’un produit hydrosoluble, indispensable chez les jeunes patients et ceux avec des épisodes récurrents d’abcès. Le diagnostic différentiel se pose avec les cancers de l’hypopharynx avec extension à la thyroïde et surinfection, les cancers de la thyroïde, les kystes infectés du tractus thyréoglosse, les adénopathies cervicales tuberculeuses [1,3,4]. Dans ces cas, l’imagerie par résonance magnétique (IRM) et la tomodensitométrie (TDM) permettent de redresser le diagnostic. Un autre diagnostic différentiel à évoquer en particulier chez le sujet jeune est la fistule du sinus piriforme. Une endoscopie pharyngée avec un transit hypopharyngo œsophagien permet dans ce cas d’objectiver respectivement l’orifice fistuleux et son trajet [4].

Le traitement habituel des abcès thyroïdiens repose sur une antibiothérapie intraveineuse associée à un drainage chirurgical [4,5]. En cas de pathologie thyroïdienne sous-jacente, l’exérèse thyroïdienne emportant l’abcès est indiquée [4]. Notre patient a été traité par tri antibiothérapie avec bonne évolution clinico biologique. Non traité, l’abcès de la thyroïde peut avoir des conséquences graves, pouvant mettre en jeu le pronostic vital. Il peut s’agir d’une rupture de l’abcès avec coulées purulentes dans le médiastin ou le péricarde, d’une fistulisation dans l’œsophage, la trachée ou à la peau, d’une thrombophlébite de la veine jugulaire interne ou d’une septicémie. On peut observer une compression du nerf récurrent entrainant une paralysie des cordes vocales, voire même la destruction du parenchyme glandulaire thyroïdien et des parathyroïdes [1,4,8]. Le traitement des tumeurs de l’œsophage cervical est essentiellement représenté par la radiochimiothérapie concomitante et la chirurgie, dont les indications font encore l’objet de controverses [7]. La radiochimiothérapie est de plus en plus proposée en première intention, la chirurgie étant réservée aux situations de récidives ou de réponse partielle à la radiochimiothérapie, le but étant d’éviter au maximum une chirurgie mutilante. Du fait de la moindre longueur de l’œsophage cervical et de la nécessité de réaliser une exérèse avec marge importante, l’intervention chirurgicale la plus réalisée est une œsophagectomie totale avec pharyngolaryngectomie totale et reconstruction de la filière digestive. La pharyngolaryngectomie totale sans œsophagectomie totale ne concerne que les lésions de la région œsophagienne cervicale haute ou hypopharyngée étendue à la bouche œsophagienne [7]. Quel que soit le type de chirurgie proposée et quel que soit le stade tumoral, le pronostic globalement sombre avec un taux de survie à 5 ans de 24% à 33% [7].

## Conclusion

L’abcès de la thyroïde est une pathologie rare mais greffée d’une morbidité importante [5]. L’imagerie joue un rôle essentiel dans le diagnostic et la détection des anomalies sous-jacentes [2]. Notre observation révèle que l’abcès thyroïdien peut être le mode de découverte d’un cancer de l’œsophage d’où l’intérêt de toujours rechercher une cause sous-jacente devant un abcès thyroïdien.

## Remerciements

A tous ceux qui ont contribué de près ou de loin à la réalisation de ce travail.
